# Using the Autofluorescence Finder on the Sony ID7000^TM^ Spectral Cell Analyzer to Identify and Unmix Multiple Highly Autofluorescent Murine Lung Populations

**DOI:** 10.3389/fbioe.2022.827987

**Published:** 2022-03-15

**Authors:** Nicholas Wanner, Jerry Barnhart, Nicholas Apostolakis, Violetta Zlojutro, Kewal Asosingh

**Affiliations:** ^1^ Asosingh Lab, Department of Inflammation and Immunity, Cleveland Clinic, Lerner Research Institute, Cleveland, OH, United States; ^2^ Sony Biotechnology Inc., San Jose, CA, United States; ^3^ Flow Cytometry Core, Cleveland Clinic, Lerner Research Institute, Cleveland, OH, United States

**Keywords:** autofluorescence, spectral flow cytometry, murine lung, Sony ID7000, autofluorescence finder

## Abstract

Autofluorescence (AF) is a feature of all cell types, though some have more than others. In tissues with complex heterogeneous cellularity, AF is frequently a source of high background, masking faint fluorescent signals and reducing the available dynamic range of detectors for detecting fluorescence signals from markers of interest in a flow cytometry panel. Pulmonary flow cytometry presents unique challenges because lung cells are heterogeneous and contain varying amounts of high AF. The goal of this study was to demonstrate how a novel AF Finder tool on the Sony ID7000™ Spectral Cell Analyzer can be used to identify and screen multiple AF subsets in complex highly AF tissues like murine lungs. In lung single cell suspensions, the AF Finder tool identified four distinct AF spectra from six highly AF subsets. The subtraction of these distinct AF spectra resulted in a resolution increase by several log decades in several fluorescent channels. The major immune and lung tissue resident cells in a murine model of asthma were easily identified in a multi-color panel using AF subtraction. The findings demonstrate the practicality of the AF Finder tool, particularly when analyzing samples with multiple AF populations of varying intensities, in order to reduce fluorescence background and increase signal resolution in spectral flow cytometry.

## Introduction

The lungs are home to a wide range of cell types that come from various lineages. This complex organ is composed of networks of various endothelial cell types, heterogeneous subsets of epithelial cells, stromal cells, and a variety of hematopoietic cells (Singer et al., 2016). An understanding of the interplay between immune and structural cells is critical in studies of pulmonary disease, such as asthma, where identifying and quantifying distinct cell populations is crucial (Tighe et al., 2019). Precise phenotyping and quantification of tissue resident cells and recruited inflammatory subsets can aid in identifying and comprehending differences between healthy and diseased subjects, particularly at the fundamental research level.

Flow cytometry has emerged as a critical tool for studying the cellular populations found in the lung due to the ease with which single cell suspensions of lung tissue can be obtained using established protocols (Reichard and Asosingh, 2019; Grant et al., 2021). Cellular autofluorescence (AF) can interfere with the signals of other fluorescent markers in flow cytometry, resulting in poor resolution or, if appropriate controls are not used, false positive results. Due to the presence of natural fluorophores such as NADH and NADPH, flavins, porphyrin, and lipofuscin, AF is an intrinsic property of all types of cells (Eldred et al., 1982; Thorell, 1983; Kiefer et al., 1998; Mayevsky and Rogatsky, 2007; Mitchell et al., 2010). AF can also occur as a result of factors in cell preparations such as activation, aldehyde fixation, and permeabilization (Cossarizza et al., 2019). Even environmental factors such as smoking have been shown to increase cell AF (Umino et al., 1999). Because of the heterogeneous nature of lung cells, which contain varying amounts of AF, flow cytometry faces unique challenges. Myeloid cells, especially eosinophils are known to have higher levels of AF due to their high intracellular flavin levels (Umino et al., 1999; Weil and Chused, 1981). Alveolar type II epithelial cells and alveolar macrophages cause severe AF in the lungs, necessitating special consideration when assessing AF in a lung panel (Maus et al., 1997; Tighe et al., 2019). Even non-viable cells can have elevated AF, which must be excluded during data analysis to avoid poor resolution of other markers or false positive results (Cossarizza et al., 2019). Avoiding the use of fluorophores in spectral regions where AF is prevalent is one way to mitigate AF’s negative impact on flow cytometric analysis. However, with the growing number of high-parameter panels to simultaneously study a large number of markers, it is critical to utilize the entire fluorescent spectrum.

Spectral flow cytometry can be used to determine the complete spectral emission signatures of single-color controls, multicolor samples, and the AF of unstained samples (Nolan and Condello, 2013). The Sony ID7000™ Spectral Cell Analyzer includes tools such as the AF Finder which enables the identification and characterization of multiple cell types’ AF signatures within a complex mixture of cells. Once these distinct autofluorescent spectra are identified, they are treated like any other fluorescent signature and mathematically separated from one another and other fluorescent signatures. This increases resolution and reduces the likelihood of false positives in the highest-AF spectral regions. We describe the AF Finder tool in the Sony ID7000 Spectral Cell Analyzer and demonstrate its ability to improve the resolution of highly AF cells using murine lung samples as an example.

## Materials and Methods

### House Dust Mite Model of Asthma

House dust mite extract (HDME) induced severe asthma as described (Herjan et al., 2018; Ouyang et al., 2020). Female C57BL/6 mice (aged 12 weeks) were purchased from The Jackson Laboratory (Bar Harbor, ME). On day 0, mice were anesthetized by isoflurane inhalation and received a 200 µl subcutaneous sensitization of 100 µg house dust mite extract (HDME) (D.pteronyssinus, Greer Laboratories) resuspended in complete Freund’s adjuvant (CFA) (Sigma) and saline. On day 14, mice were anesthetized by isoflurane inhalation then challenged intranasally with 100 µg HDME resuspended in 50 µl of saline. On day 15, the mice were euthanized and lungs were harvested. This animal study was reviewed and approved by the Cleveland Clinic Institutional Animal Care and Use Committee.

### Tissue Processing for Single Cell Suspensions

Lung single cells were isolated from lung tissue as previously described (Reichard and Asosingh, 2019), and summarized briefly here. Lungs were perfused via the right ventricle with 10 ml of PBS warmed to 37°C. From the right lung, intermediate and inferior lobes were dissected and separated from the other lung lobes, then minced with scissors until a paste-like consistency. 1 ml of a digestive enzyme cocktail containing 10 mg/ml Dispase II (Sigma Aldrich; 14365000), 1 mg/ml Collagenase A (Sigma Aldrich; 10103578001), and 1500 KU/ml DNase I (Sigma Aldrich; D5025-150KU) was added to the lung pieces and the mixture incubated at 37°C on an orbital shaker. After 30 min the mixture was gently pipetted to disperse any remaining pieces, then incubated for an additional 15 min. Lung cells were filtered through a 40 μm cell strainer and stained with LIVE/DEAD Red, a fixable amine reactive viability dye, before a short fixation in 4% paraformaldehyde. Aliquots of cells were suspended in 10% DMSO in FBS and frozen at −80°C.

### Flow Cytometry

Aliquots of cell suspensions were removed from the freezer to thaw and washed with PBS in order to remove the DMSO. Cells were suspended in 1% BSA in PBS to distribute cells into fluorescence minus one (FMO) or fully stained samples. All samples incubated in 50 µl of Fc-block (1/50 dilution; Thermo Fisher; 14-0161-85) before 50 µl of diluted cell surface antibodies were added to the cells and incubated for 30 min at 4°C. Both the Fc-block and cell surface antibodies were diluted in BD Horizon Brilliant Stain Buffer (BD; 563794), since multiple BD Horizon Brilliant dyes were used. After cell surface staining, samples were washed to remove excess, unbound, cell surface antibodies, then permeabilized in 50 µl of saponin buffer (0.1% saponin, 1% BSA, 2% Fc block, 10% normal goat serum, in PBS). After 15 min, 50 µl of diluted intracellular antibodies were added and incubated for 30 min at 4°C. Samples were washed with saponin buffer to remove excess, unbound, intracellular antibodies. Data was acquired using the Sony ID7000™ Spectral Cell Analyzer equipped with 5 lasers (355 nm, 405 nm, 488 nm, 561 nm, 637 nm). The unmixing of samples was done using the Sony ID7000™ software (v1.1.10), then unmixed FCS files were exported and opened in FlowJo v10.7.1 to create plots.

### Panel Development

The development and optimization of the multicolor panel was based on the processes described in OMIP-069 (Park et al., 2020). Based on commercial availability, antibody and fluorochrome combinations were selected by considering antigen classification, antigen co-expression, fluorochrome brightness and similarity to other fluorochromes. One antibody (von Willebrand Factor) was conjugated to a fluorochrome (Alexa Fluor 647) in-house using the Lightning-Link^®^ Rapid Alexa Fluor 647 Antibody Labeling Kit (Novus). All antibody information can be found in Table 1. Once the panel was established, cell surface and intracellular antibodies were titrated to determine optimal staining concentrations. After titrating all antibodies, staining profiles of single antibodies were compared to the staining profile in the multicolor sample. If the pattern in the single color sample did not match the pattern in the single color sample, the antibody dilution determined by titration for the multicolor sample was adjusted until the patterns in the single color and multicolor samples matched. With the final dilutions of antibodies set, FMO controls were made to define the gating boundaries during analysis. FMOs were included in each staining for antibodies where a clear separation between the positive and negative populations is not observed.

**TABLE 1:** Antibodies used in spectral flow cytometry panel.

Name	Alternative name	Clone	Fluorochrome	Source	Catalog number	Dilution by titration	Dilution by SC vs. MC
VWF	Von Willebrand Factor	Polyclonal	AF647	Dako/Novus	A0082/336-0005	1/400	1/300
CD90.2	Thy-1.2	53–2.1	BV421	BioLegend	140327	1/800	1/800
α-SMA	ACTA2	1A4	APC	Biotium	BNCA0665-250	1/800	1/800
MUC2	Mucin 2	996/1	PE	Novus	NB120-11197PE	1/200	1/75
Ly-6G	Lymphocyte antigen 6 complex	1A8	AF532	Novus	NBP2-53131AF532	1/800	1/800
Siglec-F	Siglec-5	E50-2440	BV750	BD	747316	1/400	1/400
CD11c	ITGAX	N418	BB700	BD	745852	1/400	1/400
CD24	Heat stable antigen	M1/69	BUV737	BD	612832	1/400	1/100
CD11b	ITGAM	M1/70	BV480	BD	566149	1/400	1/400
Collagen-1	Collagen type 1α	Polyclonal	FITC	Rockland	600-402-103	1/200	1/100
CD146	S-endo 1	ME-9F1	BUV563	BD	741443	1/400	1/400
PDGFRβ	CD140b	REA363	APC-Vio770	Miltenyi	130-105-119	1/200	1/200
EPCAM	CD326	G8.8	BV605	BioLegend	118227	1/200	1/200
C-kit	CD117	2B8	BV785	BioLegend	105841	1/400	1/400
VEGFR2	FLK-1; CD309	Avas12a1	BUV661	BD	741571	1/200	1/200
MHC II	M5/114	M5/114.15.2	BUV805	BD	748844	1/200	1/150
CD64	Fcgr1	X54-5/7.1	BV650	BD	740622	1/200	1/200
CD45	Leukocyte common antigen	30-F11	BV570	BioLegend	103136	1/400	1/400
Sca-1	Ly-6a/e	D7	BUV395	BD	563990	1/400	1/300
Viability Dye	N/A	N/A	Live/Dead Red	Thermo Fisher	L23102	1/16000	1/16000

## Results

### Unmixing and AF Subtraction

**FIGURE 1:**
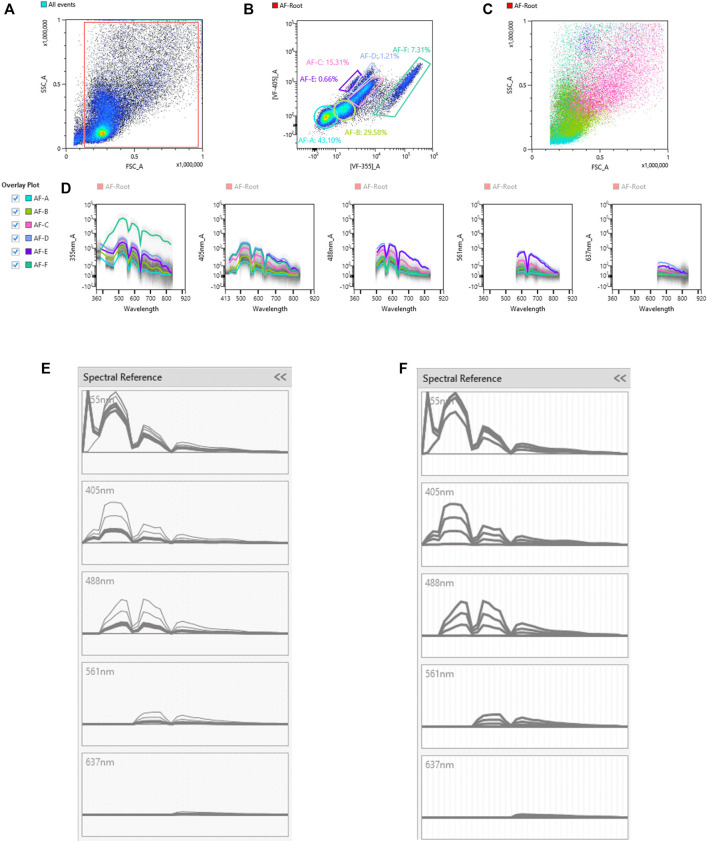
Identification of unique autofluorescent populations in an unstained sample using autofluorescence finder tool. The unstained sample with no unmixing applied was loaded into the autofluorescence (AF) Finder tool. The AF-Root gate was created to distinguish events used in the analysis from the debris **(A)**. Virtual filters in the violet range (VF-405; *y*-axis) and ultraviolet range (VF-355; *x*-axis) were used to view the events from the AF-Root gate. Populations that separate were gated as possibly unique AF populations **(B)**. The scatter plots **(C)** as well as spectral traces **(D)** for these AF populations were displayed. The spectral trace for each AF population was overlaid and evaluated to determine which AF populations were unique. The spectral traces for populations A, B, and C [bolded traces in **(panel E)**] were determined to be nearly identical. Since these traces are the same, only one of these populations was used in the spectral unmixing. Populations A, D, E, F [bolded traces in **(panel F)**] were unique AF spectra and each was used in the spectral unmixing.

**FIGURE 2:**
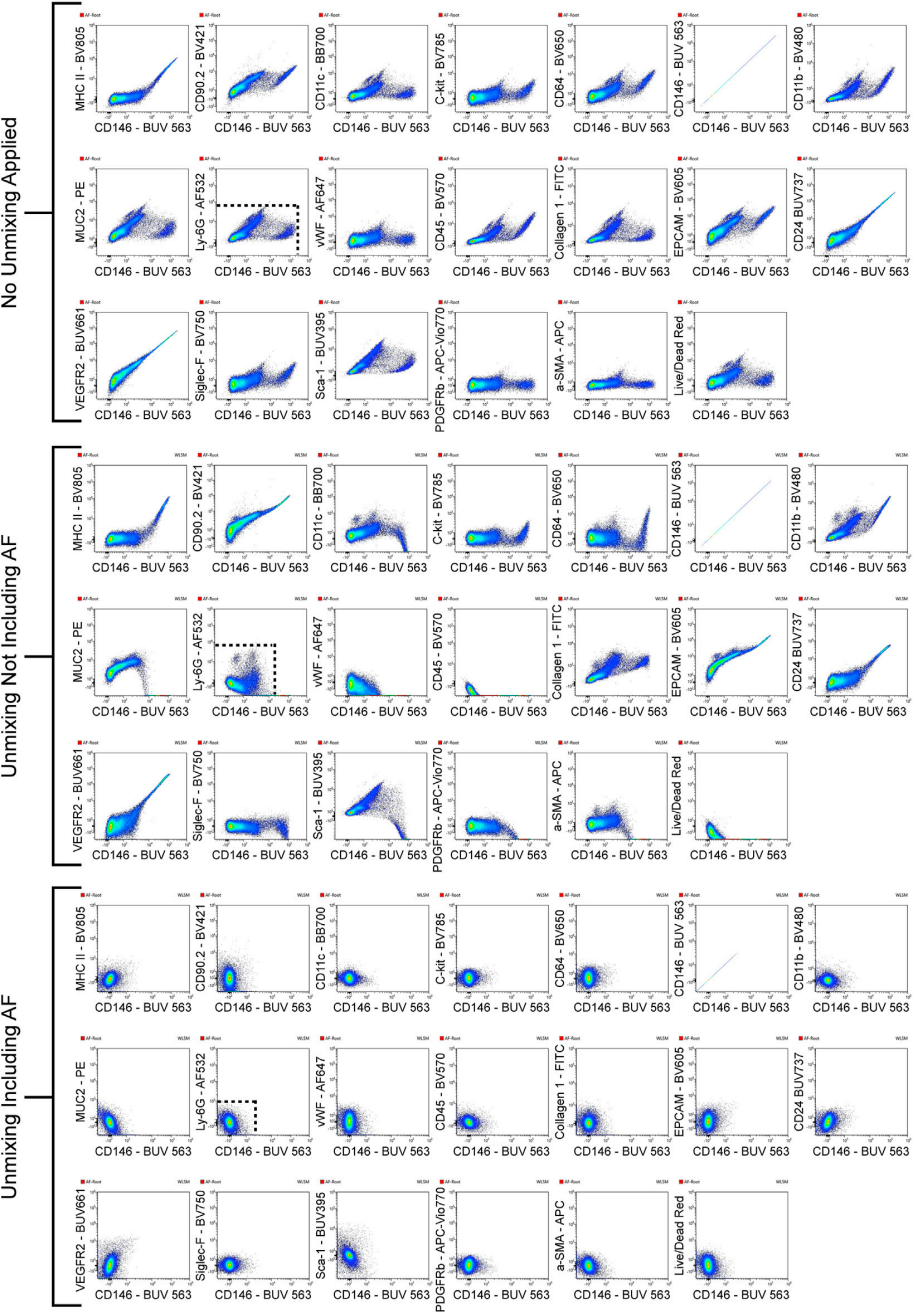
Improved resolution of unstained sample with AF included in unmixing. The unstained sample was viewed with CD146-BUV563 on the *x*-axis vs. every fluorescent parameter in the panel on the *y*-axes. Three sets of these plots are shown: the unstained sample with no unmixing applied, the unstained sample with unmixing applied but no AF populations included in the unmixing, and the unstained sample with unmixing applied and each unique AF population (AF-A, AF-D, AF-E, and AF-F) included in the unmixing. Dotted lines in the CD146-BUV563 vs. Ly-6G–AF532 plot shows how the resolution improves with unmixing and unmixing with AF included. From the plots with no unmixing applied to the plots with unmixing applied and AF included, several decades in the dynamic range are preserved.

Unique spectral signatures for each fluorochrome were used to mathematically unmix each color from another in the multicolor samples. In addition to the spectral signature from each fluorochrome, AF was also identified, and once identified, unmixed from spectral signatures of other fluorochromes. The signal from the unstained sample was recorded using the ID7000 spectral cell analyzer, then the AF Finder tool was used to identify the various AF populations. Debris was removed by gating on a scatter plot (Figure 1A), then events were viewed based on virtual filters in the ultraviolet and violet ranges where AF is typically the greatest. Virtual filters represent specific channels from the detector array. Data from these channels were displayed on dot plots when no unmixing is applied. The virtual filters were moved to different spectral regions in the software to help visualize the autofluorescent signal at different wavelengths. The virtual filters were adjusted across different wavelength ranges from different laser lines to find the spectral region with the highest intensity autofluorescent signals. Using a dot plot comparing the highest AF signal from the UV laser, and the highest AF signal from the violet laser, we were able to draw gates around any autofluorescent cluster of cells. The goal is to identify as many autofluorescent cell clusters as possible. Using this methodology, six AF populations were identified and gated (Figure 1B). A spectral reference for each of these populations was created which was viewed as a spectral trace. During the creation of the spectral reference, the software averages the spectral trace of each gated population. This average spectral intensity across all detected wavelengths is then converted from logarithmic to linear scale and the intensity is normalized in the software. It is critical to avoid having redundant AF spectral signatures, as this causes unnecessary data spread. Spectral references or unique combinations of spectral references were used to look at how autofluorescence changed the data. This was utilized to see how different AF combinations affected the unmixing of the unstained sample. If the right combination is obtained, the populations will be shown as concise double negative populations in channels where greater AF signals are expected, such as BUV395, or BV480. If redundant spectra are used together, additional spreading of the unstained populations is observed. If there are AF signatures that are unaccounted for, sub-populations that show up as dim or brightly “positive” in these channels in the unstained sample are observed. It is important to do this empirical test of applying the different combinations of AF signatures back to the unstained sample. Looking at the raw spectral traces for the autofluorescence can be misleading because it is possible to have the same spectral distribution at significantly different intensities, or different spectral patterns at similar intensities. The latter scenario can be exacerbated by the biexponential scaling, which makes it harder to identify subtle differences in intensity. Only by calculating the reference spectra for the individual traces in order to identify similarities and differences, then applying the spectra back to the unstained control, can proper identification of all of the AF signatures and elimination of duplicate signatures be ensured. AF signatures A, B, and C were nearly identical, so only one of them was needed in the unmixing (Figure 1E). AF signatures B and C were deleted from the parameter list. AF signatures A, D, E, and F all showed unique spectral waveforms and were accounted for as individual and unique autofluorescent spectra (Figure 1F). The differences in the way the spectra look from Figure 1D to Figures 1E,F is due to the conversion of the scale from log to linear, and the normalization of the intensities of the various spectra. These were the AF signatures used in the unmixing. Figure 2 shows a comparison of the unstained sample when no unmixing was applied, when unmixing was applied but no AF signatures were used, and when unmixing was applied and AF signatures were used. Most channels gained multiple log decades in resolution after subtracting the AF from the subsets identified above.

**FIGURE 3:**
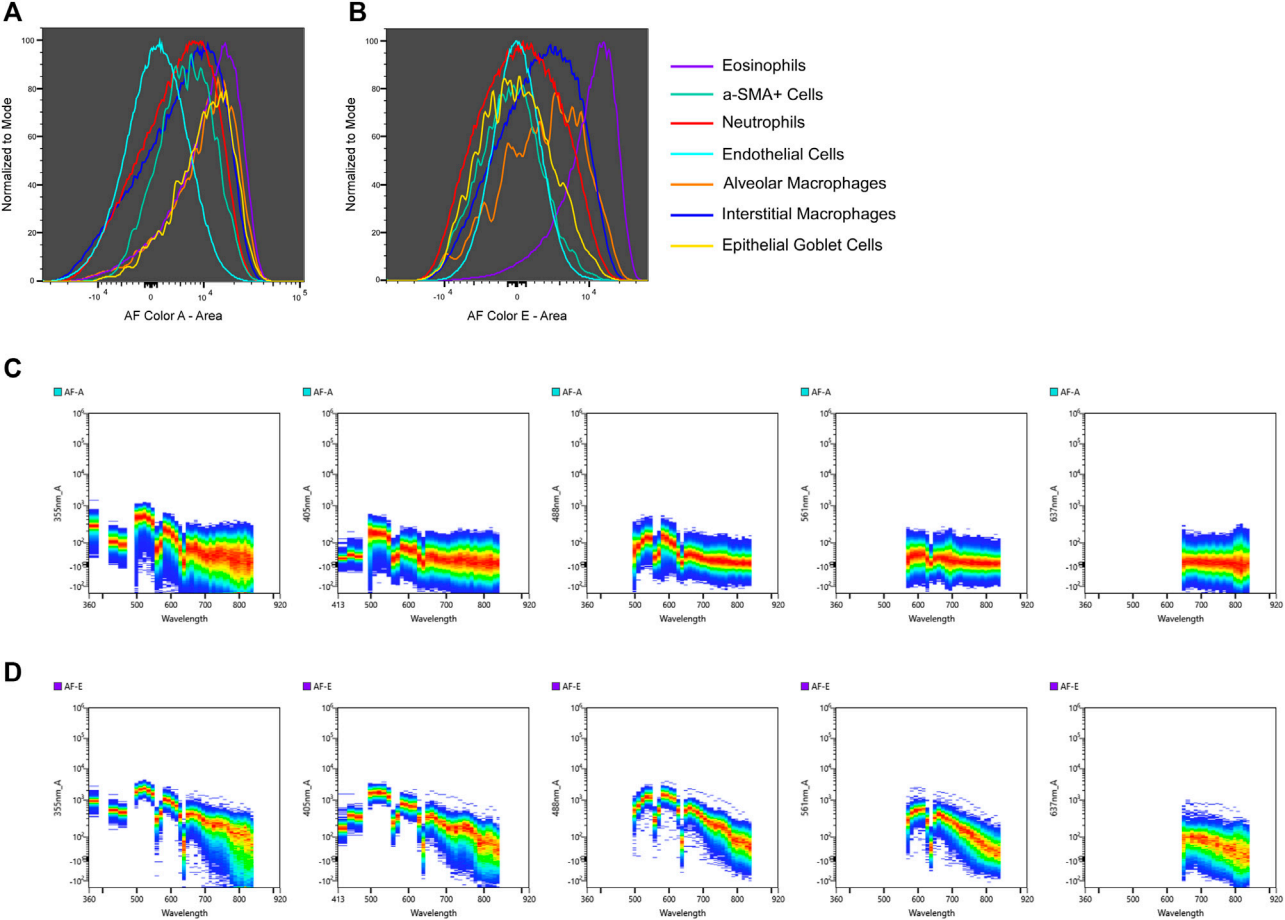
AF signature tracing to a specific cell type. The intensities of two unique AF signatures, AF color A **(A)** and AF color E **(B)**, are shown for cell types identified by immunophenotyping. AF color E is more intense in eosinophils compared to other cell types. Spectral plots of the unstained sample for AF color A **(C)** and AF color E **(D)** are shown to compare in which regions of the spectrum these AF signatures emit [wavelength (*x*-axis) is in nm]. AF color E has a distinct emission in the yellow-green region of the spectrum (500–550 nm) which is indicative of flavins. The intense presence of AF color E in eosinophils and AF color E having emission in the spectral region of flavins links AF color E to eosinophils.

In some instances AF signatures can be traced to a specific cell type. An example of AF tracing is detailed in Figure 3 which shows the intensities of AF color A (Figure 3A) and AF color E (Figure 3B) for specific cell types. Not much distinction can be made between cell types when looking at the presence of AF color A, but eosinophils display a more intense presence of AF color E compared to other cell types. The spectral plots of the unstained sample for AF color A (Figure 3C) and AF color E (Figure 3D) are shown [wavelength (*x*-axis) is in nanometers (nm)]. AF color E has the highest emission in the yellow-green range (500–550 nm), which is characteristic of flavins (Surre et al., 2018). It is established that flavin, in the form of flavin adenine dinucleotide (FAD), is present in eosinophils and is attributed to the high AF of eosinophils (Mayeno et al., 1992). The presence of an intense emission in the yellow-green range in AF color E and a strong presence of AF color E in eosinophils links AF color E to eosinophils. To confirm the correlation between AF color E and eosinophils, the intense AF color E population was gated in the multicolor sample. Events from this population were then gated based on the expression of markers in the panel to determine their cell type. This intense AF color E population was 79.8% eosinophils. Supplementary Figure S1 shows the gating described.

### Characterization of Lung Cell Subsets

**FIGURE 4:**
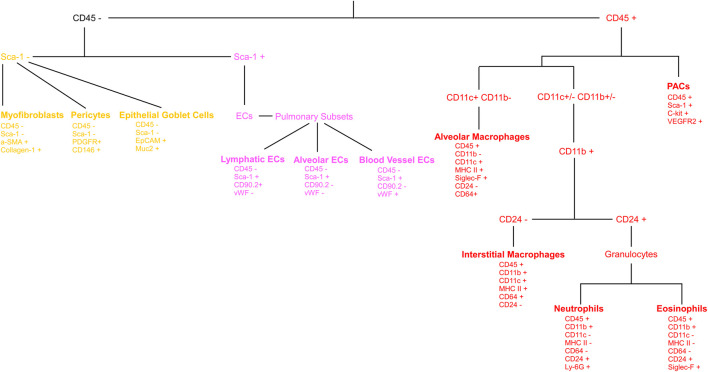
Gating tree for lung cell phenotyping. The gating hierarchy for cell populations from the lung is shown. The expression or absence of defining markers are presented under each cell type. From the CD45^+^ cells: PACs, alveolar macrophages, interstitial macrophages, neutrophils and eosinophils are identified. CD45^−^cells are further divided into CD45-/Sca-1- and CD45-/Sca-1+ groupings. From the CD45-/Sca-1- group: myofibroblasts, pericytes, and epithelial goblet cells are identified. From the CD45-/Sca-1+ group: lymphatic endothelial cells (ECs), alveolar ECs, and blood vessel ECs are identified.

**FIGURE 5:**
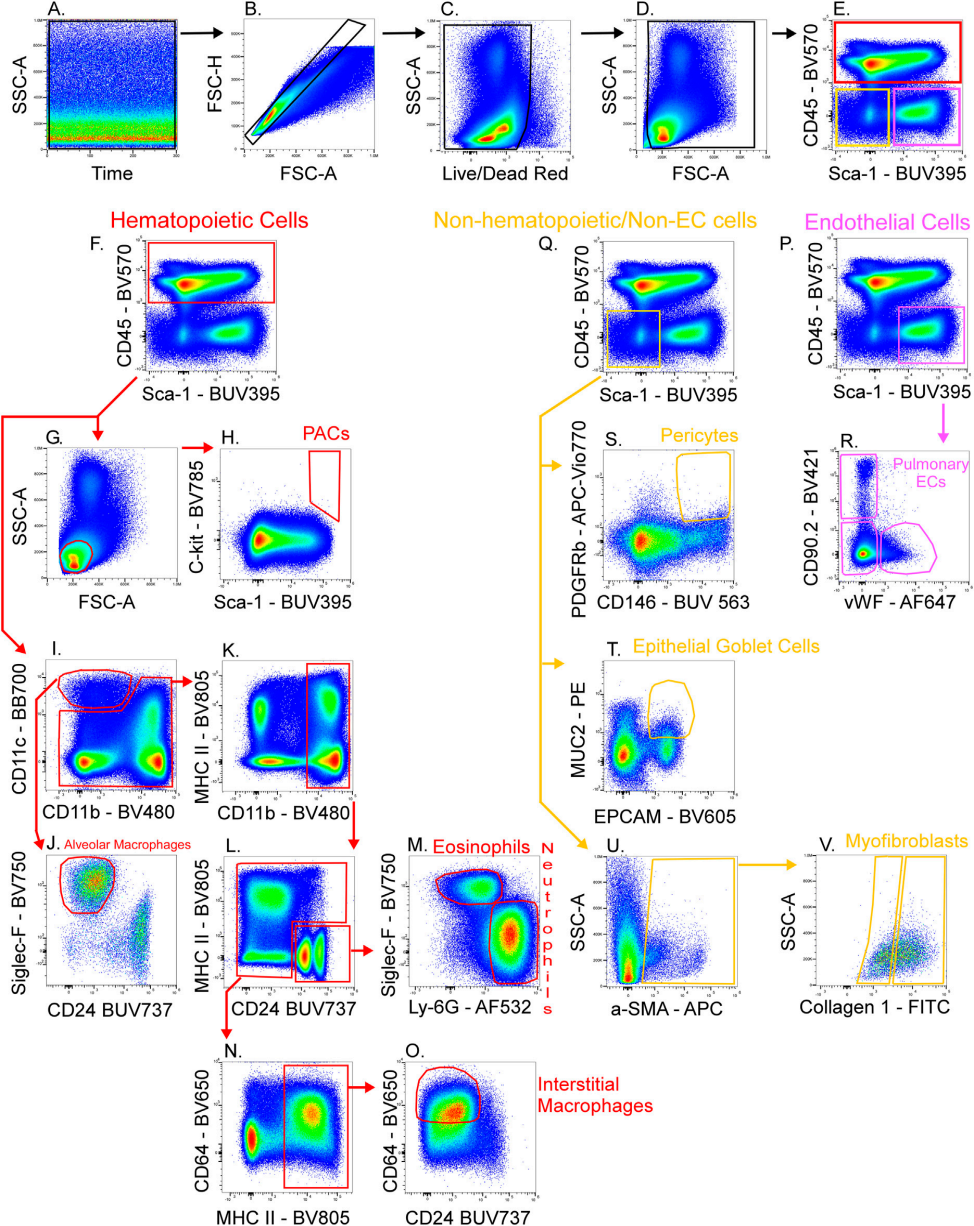
Gating strategy for lung cell phenotyping. The fully stained sample was unmixed with AF included in the ID7000 software, then FCS files were exported and opened in FlowJo. Sequential gating was used to identify cell populations. Data clean-up included time gating **(A)**, doublet exclusion **(B)**, viability gating **(C)**, and debris removal **(D)**. CD45 and Sca-1 were used to discriminate 3 populations **(E)**: hematopoietic cells (CD45^+^; red gate), endothelial cells (ECs) (CD45^−^/Sca-1^+^; pink gate), and non-hematopoietic/non-EC cells (CD45^−^/Sca-1^−^; yellow gate). A scatter plot **(G)** and expression of C-kit and Sca-1 defined proangiogenic cells (PACs) **(H)**. CD11c^+^/CD11b^−^ cells were gated **(I)** then expression of Siglec-F and absence of CD24 was used to gate alveolar macrophages **(J)**. CD11b^+^ cells **(K)** were gated from the remaining cells in plot I. CD24 was expressed on granulocytes **(L)** then Siglec-F^+^ eosinophils and Ly-6G^+^ neutrophils were gated **(M)**. The remaining cells in plot L were selected and MHC II^+^ cells were gated **(N)**. CD64^+^/CD24^−^ cells were gated as interstitial macrophages **(O)**. From the EC gate (pink gate in plot **(P)**], CD90.2 and VWF were used to gate pulmonary EC subsets as alveolar ECs (CD90.2-/VWF-), lymphatic ECs (CD90.2+/VWF-), or blood vessel ECs (CD90.2-/VWF+) **(R)**. From the non-hematopoietic/non-EC subset (yellow gate in plot **(Q)**, α-SMA^+^ cells were gated **(U)** then myofibroblasts expressing low or high amounts of collagen-1 were gated **(V)**. Also from the non-hematopoietic/non-EC subset, PDGFRb^+^/CD146^+^ pericytes **(S)**, and MUC2^+^/EPCAM^+^ epithelial goblet cells **(T)** were gated.

**FIGURE 6:**
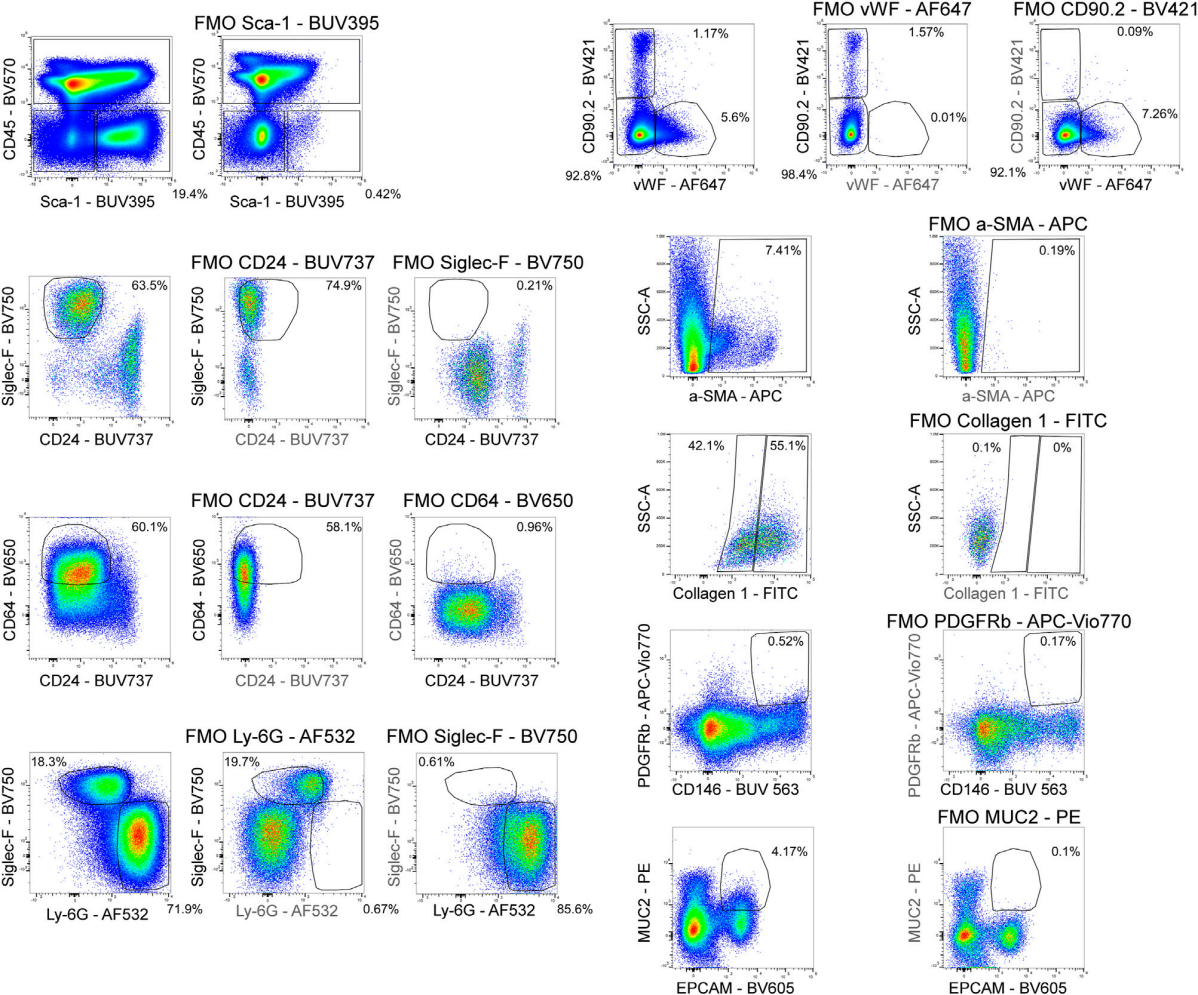
Fluorescence minus one controls. Fluorescence minus one (FMO) controls were used to set gating boundaries in the fully stained sample for parameters where the separation between negative and positive events was not clear or when the expression of a parameter was low.

**FIGURE 7:**
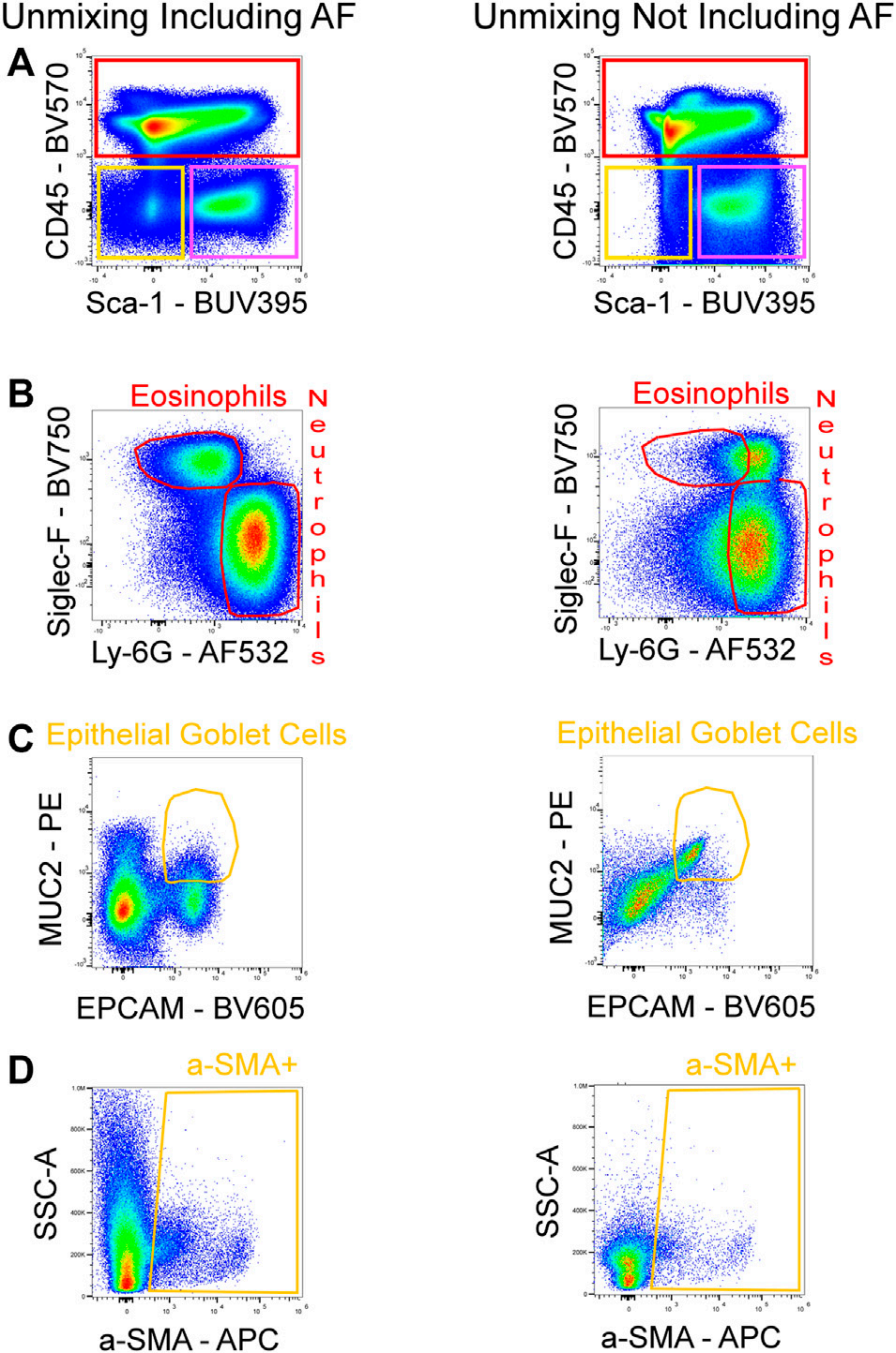
Improved resolution of fully stained sample with AF included in unmixing. AF was either included or not included in the unmixing of the fully stained sample. By including the AF in the unmixing, the resolution for BUV395 was enhanced, enabling for better detection of the non-hematopoietic/non-EC fraction (CD45-/Sca-1-; yellow gate) **(A)**. Increased resolution for AF532 was also seen, allowing for the detection of eosinophils **(B)**. The absence of AF in the unmixing masked the signals of PE and BV605 which reduced the resolution between these fluorophores and lead to false positives of epithelial goblet cells **(C)**. The poor identification of the non-hematopoietic/non-EC subset in **(A)** resulted in the loss of events downstream in the gating strategy as seen by a reduced α-SMA + population **(D)**.

Figure 4 shows a schematic gating tree for each cell population identified. Once unmixing, including AF subtraction had been applied to samples, individual cell populations were gated (Figure 5). Before sequential gating, artifacts and dead cells were excluded. Time gating (Figure 5A) selects events acquired in the flow cell during laminar flow. Fluidic disturbances can be a source of false signals because the sample stream becomes too wide for the laser beam to catch entire cells. When two or more cells travel through the laser at the same time, a pulse with the same forward-scatter height (FSC-H) as a single cell but a greater breadth and area is produced. The incidents involving a larger area are excluded from the analysis (Figure 5B). Dead cells retain a higher concentration of the amine reactive viability dye and are therefore eliminated from analysis (Figure 5C). Cell debris has relatively low side-scatter and forward scatter, which allows it to be distinguished from intact cells (Figure 5D). If the light scatter profile cannot clearly distinguish between debris and complete cells, a nuclear dye can be applied to help. Expression of CD45 and Sca-1 divided gating into 3 subsets: hematopoietic cells (CD45^+^), endothelial cells (ECs) (CD45-/Sca-1+) (Kotton et al., 2003), and non-hematopoietic/non-EC cells (CD45-/Sca-1-) (Figure 5E). The gating strategy utilized in identifying populations from the hematopoietic subset was adapted from a previous paper (Misharin et al., 2013). To gate myeloid cells, cells from the CD45 ^+^ gate (Figure 5F) were selected. Alveolar macrophages were gated as CD11b- and CD11c+ (Figure 5I), then expression of Siglec-F and absence of CD24 identified their final gate (Figure 5J) (Rose et al., 2010). Interstitial macrophages express CD11b (Figure 5K), MHCII (Figure 5N), and CD64, but do not express CD24 (Figure 5O). The interstitial macrophage gate was confirmed by expression of CD11c (not shown). Although we did not gate for dendritic cells (DC), when gating for interstitial macrophages on the CD64 vs. CD24 plot the CD11b + DC population was not prominent in our samples. This could be due to differences in tissue digestion or disease model. CD24 is expressed on granulocytes (Figure 5L) which were further classified as eosinophils and neutrophils based on expression of Siglec-F and Ly-6G respectively (Figure 5M). Hematopoietic proangiogenic cells (PAC) are C-kit+/sca-1+/VEGFR2+ (Figure 5H) (Rose et al., 2015). The presence of C-kit + Sca-1+ cells was too low to identify VEGFR2 expression in this subset. CD45^−^cells were further characterized as endothelial cells (Figure 5P) or non-hematopoietic/non-EC cells (Figure 5Q) based on expression or absence of Sca-1. Expression of CD90.2 and VWF further defined ECs as alveolar ECs (CD90.2-/VWF-), lymphatic ECs (CD90.2+/VWF-), or blood vessel ECs (CD90.2-/VWF+) (Figure 5R) (Grant et al., 2021). Populations in the non-hematopoietic/non-EC subset were then defined as pericytes (PDGFRb+/CD146+) (Figure 5S) (Hung et al., 2013) and epithelial goblet cells (MUC2+/EPCAM+) (Figure 5T) (Tawiah et al., 2018). Myofibroblasts expressing low or high amounts of collagen-1 (Figure 5V) were identified after selecting for α-SMA + cells (Figure 5U) (Reichard et al., 2018). When antigen expression was unclear, fluorescence minus one (FMO) controls were utilized to set gates (Figure 6). The fully stained sample, as well as the FMO control(s) used to establish gates, are shown. The frequencies of gated events are displayed. In addition to the improvement in resolution of the unstained sample shown in Figure 2, including AF in the unmixing improved resolution of several subsets in the fully stained sample. Figure 7 shows a comparison of plots of the fully stained sample where AF was either included or not included in the unmixing. Not including AF in the unmixing resulted in the loss of several cell populations including the non-hematopoietic/non-EC subset (Figure 7A) and eosinophils (Figure 7B).

## Discussion

In recent years, spectral flow cytometry technology has advanced significantly and now offers several substantial advantages over conventional flow cytometry (Robinson and Roederer, 2015; Robinson, 2019). Until recently, conventional flow cytometers collected data by utilizing a narrow band of the optical spectrum. While conventional flow cytometry is an extremely useful technique that has been widely used for decades (Vembadi et al., 2019), spectral flow cytometry measures the entire fluorescence emission spectrum of each cell, allowing for the easy separation of complex mixtures of fluorochrome combinations that are difficult to detect using conventional flow cytometry (Sahir et al., 2020; Solomon et al., 2020). As a result, in contrast to traditional compensation, spectral unmixing provides optimal resolution of dimly fluorescent subsets because all fluorescent signals, not just peak emission, are collected (Futamura et al., 2015). The ability to collect fluorescence across the entire spectrum allows for detailed profiling of AF using unstained cells. Autofluorescence, like any fluorochrome, produces a unique spectral signature that can be used during spectral unmixing to subtract the AF background from a fully stained panel (Schmutz et al., 2017; Niewold et al., 2020). This differs from a common practice in conventional flow cytometry to address AF which is avoiding areas in the spectrum where AF is customary (Garn, 2006). For these reasons, the primary characteristics of spectral flow cytometry, increased fluorescence collection and reduced AF background, enable unprecedented signal resolution.

Multiple AF populations with distinct spectral signatures were identified and included in the spectral unmixing of a high-parameter flow cytometry panel using the novel AF finder tool available on the Sony ID7000™ spectral cell analyzer. This enabled the identification of specific cell types across a variety of cellular subsets found in a murine lung exacerbated asthmatic model. AF subtraction from multiple populations in the spectral unmixing greatly improved the fluorescence resolution in several channels.

Including AF in spectral unmixing is not a novel idea, and it has been demonstrated to improve the resolution between positive and negative populations in highly AF samples (Niewold et al., 2020). The unstained sample is typically treated as a single AF population and included in the unmixing. While this is an important step toward improving data quality in highly AF samples, it does not allow for the isolation of multiple and distinct AF signals in a sample. This is especially important in heterogeneous samples containing a variety of cell types. The recognition of multiple AF populations found in samples is becoming more important as spectral cytometry becomes more widely utilized. In most cases, AF signal is avoided or reduced, but in some cases, AF is used to identify and isolate cell types (Dorward et al., 2013). The AF finder tool was used to identify distinct AF signatures, and an example of how these AF signatures can be tracked back to specific cell types was provided. Biomolecules with high AF that are found in cells can glow in certain parts of the spectrum, indicating their presence in the AF signature. Cell types that contain these biomolecules are linked to the AF signature, and immunophenotyping the AF population validates their identity. Whether the goal is to extract AF signal to improve resolution or to use AF to characterize a cell type, the ID7000’s AF finder makes identifying distinct AF populations simple and routine for any sample type.

## Data Availability

The original contributions presented in the study are included in the article/Supplementary Material, further inquiries can be directed to the corresponding author.
