# Supplementary material for: Using the Autofluorescence Finder on the Sony ID7000TM Spectral Cell Analyzer to Identify and Unmix Multiple Highly Autofluorescent Murine Lung Populations
Source: Front Bioeng Biotechnol. 2022 Mar 15;10:827987. doi: 10.3389/fbioe.2022.827987 (PMC8965042; doi:10.3389/fbioe.2022.827987)
Supplement: Supplementary file 1 [file Image1.pdf]

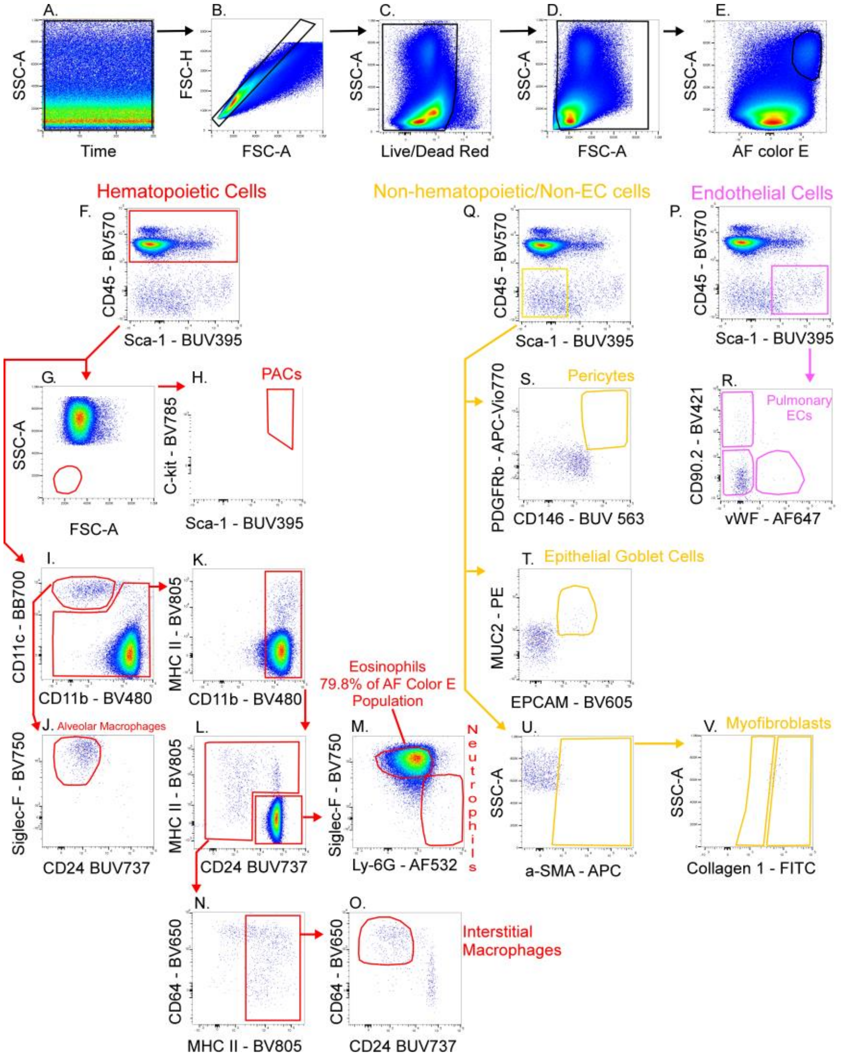

Supplemental Figure 1. Gating of AF color E for eosinophil confirmation.

The same aggregate correction was followed as in figure 5. The intense AF color E population (E) was gated then followed the same gating strategy as in figure 5. As seen in the plots, the majority of cells from the AF color E population were gated as eosinophils.
